# Evaluating risk factors and complications in mandibular ramus block grafting: a retrospective cohort study

**DOI:** 10.1007/s00784-024-05613-6

**Published:** 2024-03-22

**Authors:** Ferit Bayram, Gökhan Göçmen, Yaşar Özkan

**Affiliations:** 1 Department of Oral and Maxillofacial Surgery, Marmara University School of Dentistry, Istanbul, 34854 Turkey; 2 Department of Oral and Maxillofacial Surgery, Faculty of Dentistry, University of Marmara, Basibuyuk Yolu 9/3 34854 Basibuyuk / Maltepe / Istanbul, Istanbul, 34854 Turkey

**Keywords:** Alveolar ridge augmentation, Bone transplantation, Retrospective studies, Dental implants, Alveolar bone grafting

## Abstract

**Objectives:**

This retrospective cohort study aimed to identify the complications and risk factors associated with alveolar grafting using autologous mandibular ramus grafts, guided by the research question: What are the complications encountered in patients undergoing alveolar bone grafting using autologous mandibular ramus block and what are the risk factors associated with the development of these complications?

**Materials and methods:**

The study included 70 patients who underwent alveolar crest augmentation with autologous mandibular ramus block grafting. Intraoperative, early postoperative, and late postoperative complications were analyzed, as were various risk factors.

**Results:**

The results showed that the majority of patients had successful outcomes with minimal complications. Sex was found to significantly influence the visibility of the inferior alveolar nerve (IAN). Early postoperative complications were associated with IAN visibility and the use of a single screw for graft fixation. Late postoperative complications were significantly associated with the presence of infection.

**Conclusion:**

The findings emphasize the importance of careful surgical techniques, infection prevention, and patient selection in minimizing complications.

**Clinical relevance:**

This article may contribute to clinicians’ and so patients’ understanding of potential risk factors associated with over all ramus block grafting procedure. Based on this information, clinicians can also improve their ability to manage risk factors and associated complications and compare ramus block grafting with other alternatives to determine the best treatment approach for that particular patient.

## Introduction

The demand for immediately obtaining both cosmetic and functional results in implant-supported prosthetic rehabilitations has gained significant popularity. The concept of same-day implant placement and prosthesis placement is highly sought after in contemporary clinical practice. Graftless solutions are frequently favored by both patients and clinicians [1]. Therefore, patients who are persuaded to endure extended waiting periods and undergo supplementary grafting procedures may find it challenging to tolerate complications that might be encountered.

Augmentation with a block graft from the ramus is accepted as the gold standard for alveolar bone augmentation because of both the complications encountered at the donor site and the results obtained on the recipient side [2]. Chang et al. described the ideal donor site as one that allows for easy harvesting, minimal bleeding, straightforward postoperative care, the possibility of reharvesting, rapid wound healing, and minimal side effects [3]. This procedure is renowned for its predictable and successful outcomes, with ‘early-phase’ complications identified as those occurring within the first eight weeks after surgery, and ‘late-phase’ complications as those emerging beyond the initial eight-week period [2].

Research on autologous bone procedures is extensive, yet the majority of studies have focused primarily on reconstructive procedures or complications related to harvesting rather than on potential risk factors that could affect surgical outcomes [4–7]. However, considering that autogenous grafting operations are elective surgeries, surgeons must balance the potential benefits against the risks associated with the operation for their patients. Additionally, the practitioner’s firm understanding and ability to communicate the spectrum and frequency of possible complications [8] should be an integral part of the consent and treatment planning process [9].

Complications associated with autologous bone augmentation procedures can occur during surgery, as well as in the early or late healing phases, and may manifest in both the augmented recipient site and the harvested donor area [10, 11]. Previous studies have identified pain, swelling, bleeding, infection, mucosal dehiscence, limited mouth opening, changes in the contour of the donor area, and temporary or permanent neurosensory disturbances in the inferior alveolar nerve as the most frequently reported complications following intraoral autogenous bone graft harvesting [12–15]. Identifying potential risk factors for postoperative complications is the first challenge in preventing them. Hence, there is a need for clinical studies that analyze the complication rates, risk factors, and outcomes of the techniques employed in these procedures.

This study aimed to analyze the risk factors and complication rates associated with alveolar grafting procedures using the mandibular ramus as a donor site in patients with alveolar crest atrophy by evaluating data from a five-year period at a university teaching hospital. The ultimate goal of the study is to contribute clinicians to make a treatment plan considering the possible risk factors of this operation.

## Materials and methods

### Study design

This retrospective cohort study was conducted at Marmara University Faculty of Dentistry, a university teaching hospital, between January 2015 and January 2020. The records of all patients who underwent alveolar crest augmentation via autologous bone grafts from the intraoral ramus and met the inclusion criteria were reviewed. Eligible patients were identified by searching a prospectively maintained electronic hospital database. Those who met the inclusion criteria were analyzed separately. The investigators collected a detailed and standardized dataset from the patients’ medical and radiological records, covering the entire process from preautograft preparation to the completion of prosthetic treatment and including at least one year of follow-up. This dataset included medical records, surgical reports and photographs, providing a holistic view of each patient’s care experience.

Patients were eligible for participation after a minimum follow-up period of 12 months. An indicator of bone augmentation was the presence of severe alveolar crest atrophy, which was classified as Class III or IV according to Cawood and Howell’s classification [16]. The study included those for whom the mandibular ramus area was utilized as the donor site for alveolar crest reconstruction. Additionally, participants who had undergone implant surgery 4–8 months after bone augmentation and who were periodontally healthy or had well-controlled periodontal conditions were chosen.

Patients with alveolar bone defects resulting from any pathology, such as tumors, cysts, or infections, were excluded to eliminate the influence of variables arising from pathological conditions. Additionally, patients who had horizontal bone defects, had undergone alternative approaches for augmenting resorbed alveolar crests, had a history of alcohol misuse (over 14 standard drinks per week for men, 7 for women) and/or heavy smoking (10 or more cigarettes per day), or had any systemic disease affecting osteogenesis, including a history of head or neck radiotherapy, were excluded. All surgical procedures were performed by a team of three surgeons, each at a different level of experience: senior, mid-level, and junior. This classification reflects their roles within our university hospital as a professor, associate professor, and assistant professor, respectively, rather than their specific experience with the autogenous grafting procedure. Each surgeon, irrespective of their title, possesses extensive experience in this surgery, having performed it both before and throughout the study period.

### Outcome measures

The clinical characteristics, comorbidities, surgical records, adverse events, and management details of the patients were evaluated.

### Covariates

The risk factors identified for risk analysis included sex (female, male), age (continuous), medication (none, one medication, polymedication), systemic disease (ASA I, ASA II), smoking (absent, present), alcohol (absent, present), tooth gap (one, two, three), type of anesthesia (local, sedation, general), region (anterior mandible, posterior mandible, anterior maxilla, posterior maxilla), experience (senior, mid-level, junior), side (unilateral, bilateral), incision (vestibular, sulcular flap), osteotomy (piezosurgery, bur), number of screws (one, two), stabilization of bone graft prior to implant placement (good, fair, excellent), implant brand (Straumann, Megagen, Astra, Zinedent), and patient satisfaction measured on a 10-point visual analog scale (VAS).

### Complications

Complications were classified into intraoperative, early postoperative, and late postoperative categories [8]. Intraoperative complications assessed included IAN visibility (absent, present), a bad fracture (absent, present), and bleeding (absent, present). Early postoperative complications were evaluated as hematoma (absent, present), neurosensory disturbance in the 1st week (absent, present), infection (absent, present), or dehiscence (absent, present). Late postoperative complications included resorption (minor, major), screw fracture (absent, present), graft separation (absent, present), graft loss (absent, present), neurosensory disturbance in the 1st year (absent, present), and aesthetic problems (absent, present). Neurosensory changes were assessed by reviewing medical records noted during patients’ follow-up appointments, categorizing them as absent or present. Patients exhibiting at least one symptom, such as pain, swelling, redness, fever, or purulent discharge, were considered positive for infection (absent, present). Volumetric resorption of the grafted areas was estimated intraoperatively during implant placement using titanium micro screws used in the reconstructive procedure as a reference. Using a UNC15 periodontal probe, the distance between the screw head and the bone profile was measured post flap elevation on both the mesial and distal sides of each screw. This measurement was used to determine the extent to which the screw was exposed above the bone level following graft resorption. The measurements were rounded to the nearest half-millimeter, and the arithmetic mean of the mesial and distal values for each screw was recorded [16]. Complete removal of the autologous bone graft was documented as a major failure (absent, present). Failure was evaluated as graft loss and the need for reharvest autogenous bone graft or guided bone regeneration.

### Surgical procedure

The type of anesthesia used for the surgical procedure to harvest an autogenous ramus bone block graft was determined using local anesthesia or a combination of sedation/general anesthesia, depending on patient preference and the operating surgeon’s preference. An incision is made in the buccal mucosa, either via a vestibular incision or via a sulcular flap incision, to adequately expose the donor site. The desired size of the bone block was marked, and osteotomies were carefully performed using either piezosurgery or a bur to extract the graft from the mandibular ramus. After the graft is removed, the donor site is smoothed using diamond burs to eliminate any sharp bone edges that could increase the risk of dehiscence. The harvested bone was kept in sterile saline. The autologous bone graft was fixed to the recipient site using 1 or 2 titanium bone fixation screws. All sharp bone edges that could increase the risk of exposure of the bone block were softened with diamond burs. To facilitate tension-free closure of the flaps, horizontal periosteal releasing incisions were made. The wound is then closed using simple sutures, typically with 4/0 silk or 4/0 PGA, ensuring a secure and neat closure that promotes optimal healing.

Following the surgery, patients were instructed to apply cold compresses to the operated areas for the first 24 h and take prescribed oral antibiotics (amoxicillin and clavulanate) for five days. Pain was managed with NSAIDs such as ibuprofen for 3–5 days or paracetamol for those unable to take NSAIDs. Dietary restrictions included a cold, liquid diet for the initial four days, followed by the consumption of soft foods until suture removal. Twice-daily 0.2% chlorhexidine mouthwashes were advised for ten days. Sutures were removed after 14–21 days, and patients were not allowed to wear any removable prosthesis in the operated area for six weeks to prevent trauma and ensure proper healing.

Dental implants were placed over a period ranging from 4 to 8 months after augmentation. At this time, the titanium screws previously used for fixation of the autologous graft were removed. Implant placement was performed according to the closed healing method protocol. The postoperative instructions following implant surgery were similar to those of the previous surgery. Patients started prosthetic phases 3 to 6 months after implant placement. Cemented or fixed permanent prostheses were delivered to the patients as definitive prostheses.

The normality of the data was assessed using the Shapiro‒Wilk test. For normally distributed data, the mean and standard deviation (SD) were used to express the findings. In contrast, data not normally distributed are presented as the median and interquartile range. The frequencies of categorical data are expressed as numbers and percentages. The data are also represented as odds ratios (ORs) accompanied by 95% confidence intervals (CIs). To evaluate the impact of all risk factors on the occurrence of postoperative complications (absent/present), univariate logistic regression analysis was conducted.

## Results

At a university hospital in Turkey, between January 2015 and January 2020, 152 patients underwent alveolar crest augmentation with autologous bone grafts harvested from the intraoral ramus area of the mandible. Of these, 39 were excluded because the surgeries were not performed by the included surgeons, and 43 were omitted due to missing data in the surgical records. Ultimately, 70 patients (36 women and 34 men) meeting the inclusion criteria with complete clinical data were analyzed for this study.

The average age was 44.96 years, with a standard deviation (SD) of 13.56 years. In terms of medication use, a significant majority of the participants (74.29%, *n* = 52) reported not using any medication. A smaller fraction of the sample was on one medication (20.00%, *n* = 14), and a minimal number of patients reported polymedication use (5.71%, *n* = 4). Systemic health status was classified as ASA I in 70.00% (*n* = 49) of the participants, indicating no significant systemic disease, while 30.00% (*n* = 21) fell into the ASA II category, suggesting mild systemic disease. Lifestyle habits, such as smoking and alcohol use, were also recorded. A majority of participants did not smoke (62.86%, *n* = 44), while 37.14% (*n* = 26) were smokers. Alcohol consumption patterns mirrored this trend, with a large majority not consuming alcohol (84.29%, *n* = 59) and a smaller group reporting alcohol use (15.71%, *n* = 11) (Table 1).

**Table 1:** Demographic and clinical characteristics of study participants

	**n**	**%**	**Mean**	**SD**
**Age**			44.96	13.56
**Gender**				
Female	36	51.43		
Male	34	48.57		
**Medication**				
None	52	74.29		
One medication	14	20.00		
Polymedication	4	5.71		
**Systemic disease**				
ASA I	49	70.00		
ASA II	21	30.00		
**Smoking**				
Absent	44	62.86		
Present	26	37.14		
**Alcohol use**				
Absent	59	84.29		
Present	11	15.71		

Table 2 shows the intraoperative characteristics of the autogenous bone graft and dental implant surgeries. In bone graft surgeries, a distribution of surgeon experience levels was observed, with mid-level surgeons performing 40% of the procedures, followed by senior surgeons performing 34.29% and juniors performing 25.71%. The predominant type of anesthesia used was local (81.43%), with sedation and general anesthesia being less common. Unilateral surgeries were more common (78.57%) than bilateral surgeries were. The posterior mandible was the most common region operated on (40%), followed by the anterior maxilla and anterior mandible. A majority of these procedures utilized piezosurgery for osteotomy (92.86%), and in most cases, the IAN was not visible (82.86%). In the dental implant surgeries, the average bone resorption was 1.09, with a standard deviation of 0.89. Among the implant brands, Straumann was the most commonly used (47.14%), followed by Megagen and Astra. The primary stabilization achieved was categorized as good in 44.29% of the patients and excellent in 42.86%, with a smaller proportion deemed fair.

**Table 2:** Intraoperative characteristics of autogenous bone graft and dental implant surgeries

	**n**	**%**	**Mean**	**SD**
Bone graft surgery				
Surgeon				
Senior	24	34.29		
Mid-level	28	40		
Junior	18	25.71		
**Type of anesthesia**				
Local	57	81.43		
Sedation	11	15.71		
General	2	2.86		
**Side**				
Unilateral	55	78.57		
Bilateral	15	21.43		
**Region**				
Posterior mandible	28	40		
Anterior maxilla	25	35.71		
Anterior mandible	11	15.71		
Posterior maxilla	3	4.29		
Whole maxilla	2	2.86		
**Tooth gap**				
One	23	32.86		
Two	38	54.29		
Three	9	12.86		
**Osteotomy**				
Piezosurgery	65	92.86		
Bur	5	7.14		
**IAN visible**				
Absent	58	82.86		
Present	12	17.14		
**Number of screws**				
One	13	18.57		
Two	57	81.43		
**Dental implant surgery**				
**Bone resorption**			1.09	0.89
**Implant brand**				
Straumann	33	47.14		
Megagen	25	35.71		
Astra	7	10		
Zinedent	4	5.71		
Bego	1	1.43		
**Primary stabilization**				
Fair	9	12.86		
Good	31	44.29		
Excellent	30	42.86		

Table 3 shows the occurrence of various complications across different stages of surgical procedures, categorized as intraoperative, early postoperative, or late postoperative complications. During the intraoperative phase, visibility of the inferior alveolar nerve (IAN) was noted in 17.14% of the patients, while incidences of severe fracture and bleeding were both reported in 8.57% of the surgeries. In the early postoperative period, a small percentage of patients experienced hematoma (7.14%) and neurosensory disturbance within the first week (11.43%), with infection and dehiscence occurring in 18.57% and 22.86%, respectively. The late postoperative complications included varying degrees of bone resorption, with minor resorption occurring in 78.57% and major resorption occurring in 21.43% of patients. Screw fracture and graft separation were observed in 7.14% and 15.71%, respectively, of the patients, while graft loss was noted in 14.29% of the surgeries. There were no reported cases of neurosensory disturbance in the first year after surgery. Aesthetic problems were present in 17.14% of the patients.

**Table 3:** Overview of intraoperative, early postoperative, and late postoperative complications

	**n**	**%**
Intraoperative complications		
IAN visibility		
Absent	58	82.86
Present	12	17.14
**Bad fracture**		
Absent	64	91.43
Present	6	8.57
**Bleeding**		
Absent	64	91.43
Present	6	8.57
**Early postoperative complications**		
**Hematoma**		
Absent	65	92.86
Present	5	7.14
**Neurosensory disturbance, 1st week**		
Absent	62	88.57
Present	8	11.43
**Infection**		
Absent	57	81.43
Present	13	18.57
**Dehiscence**		
Absent	54	77.14
Present	16	22.86
**Late postoperative complications**		
**Resorption**		
Minor	55	78.57
Major	15	21.43
**Screw fracture**		
Absent	65	92.86
Present	5	7.14
**Graft separation**		
Absent	59	84.29
Present	11	15.71
**Graft loss**		
Absent (success)	60	85.71
Present	10	14.29
**Neurosensory disturbance, 1st year**		
Absent	70	100
Present	0	0
**Aesthetic problems**		
Absent	58	82.86
Present	12	17.14
**Complication**		
Absent	51	72.9
Present	19	27.1

Table 4 presents the univariate logistic regression analysis of various factors influencing intraoperative complications during surgical procedures. The analysis included the influence of age, sex, tooth gap, type of anesthesia, surgeon experience, surgical side, incision type, and osteotomy method on complications such as IAN visibility, bad fracture, and bleeding. Notably, sex (female) significantly impacted IAN visibility, with an odds ratio (OR) of 6.15 and a 95% confidence interval (CI) ranging from 1.46 to 42.4, indicating a greater likelihood of IAN visibility in females. However, when paresthesia was compared in the first postoperative week using Fisher’s exact test, no significant difference was found between the sexes (*p* = 0.261). Other factors, such as age, tooth gap, type of anesthesia, and bilateral surgical side, generally had no significant impact (ns) on the occurrence of IAN visibility, severe fractures, or bleeding, as evidenced by their p values and confidence intervals. For some factors, including experience (junior), we could not fit a logistic regression, possibly due to perfect separation or linear dependence of the predictors. To resolve the issues of perfect separation and multicollinearity in our logistic regression analysis, we removed the problematic variables from the model to ensure more reliable and interpretable results.

**Table 4:** Univariate logistic regression analysis of intraoperative complications

	**OR**	**95% CI**	**P**	**sig.**
IAN visibility				
Age	0.996	“0.952 to 1.05”	0.879	ns
Gender (female)	6.15	“1.46 to 42.4”	0.026	*
Tooth gap (three)	1.27	“0.165 to 6.75”	0.795	ns
Type of anesthesia	1.46	“0.343 to 6.28”	0.602	ns
Experience (junior)	Could not fit logistic regression			
Side (bilateral)	1.28	“0.255 to 5.09”	0.741	ns
Incision (sulcular flap)	1.26	“0.336 to 4.45”	0.722	ns
Osteotomy (bur)	1.23	“0.0597 to 9.35”	0.861	ns
**Bad fracture**				
Age	0.986	“0.927 to 1.05”	0.641	ns
Gender (female)	2	“0.364 to 15.2”	0.442	ns
Tooth gap (three)	Could not fit logistic regression			
Type of anesthesia (sedation)	Could not fit logistic regression			
Experience (junior)	1.38	“0.152 to 12.5”	0.762	ns
Side (bilateral)	1.96	“0.252 to 11.3”	0.464	ns
Incision (sulcular flap)	0.833	“0.110 to 4.61”	0.84	ns
Osteotomy (bur)	Could not fit logistic regression			
Ian visibility (present)	2.7	“0.342 to 15.9”	0.287	ns
**Bleeding**				
Age	0.991	“0.933 to 1.06”	0.782	ns
Gender (female)	0.939	“0.163 to 5.41”	0.942	ns
Tooth gap (three)	Could not fit logistic regression			
Type of anesthesia	Could not fit logistic regression			
Experience (junior)	2.88	“0.255 to 65.0”	0.405	ns
Side (bilateral)	Could not fit logistic regression			
Incision (sulcular flap)	0.833	“0.110 to 4.61”	0.84	ns
Osteotomy (bur)	Could not fit logistic regression			
Ian visibility (present)	2.7	“0.342 to 15.9”	0.287	ns
Bad fracture	Could not fit logistic regression			

Table 5 presents a comprehensive univariate logistic regression analysis aimed at predicting early postoperative complications. The analysis encompasses a range of factors, including age, sex, medication usage, systemic disease status (ASA II), smoking and alcohol consumption habits, tooth gap, type of anesthesia, surgical region, surgeon’s experience, surgical side, incision type, osteotomy method, and the number of screws used. The identification of certain variables, such as sex and hematoma status, could not be effectively performed. This limitation is attributed to the potential issues of perfect separation or linear dependence of predictors. For the variables successfully analyzed, the odds ratios (ORs) and 95% confidence intervals (CIs) provided insights, although many did not show statistically significant associations with early postoperative complications. However, several notable findings emerged: the presence of IAN visibility was significantly associated with an increased likelihood of hematoma (OR 9.33, *p* = 0.023) and early neurosensory disturbance in the first week (OR 28, *p* < 0.001). Additionally, using a single screw (OR 6.12, *p* = 0.008) appeared to significantly elevate the risk of infection. Furthermore, infection itself was a significant predictor of dehiscence (OR 6.22, *p* = 0.006).

**Table 5:** Logistic regression analysis for predicting on early postoperative complications

	**OR**	**95% CI**	**P**	**sig.**
Hematoma				
Age	0.985	“0.922 to 1.06”	0.66	ns
Gender (female)	Could not fit logistic regression			
Medication	Could not fit logistic regression			
Systemic disease (ASA II)	0.563	“0.0278 to 4.11”	0.617	ns
Smoking (present)	2.74	“0.425 to 21.9”	0.288	ns
Alcohol (present)	1.38	“0.0666 to 10.6”	0.785	ns
Tooth gap (three)	Could not fit logistic regression			
Type of anesthesia (sedation)	Could not fit logistic regression			
Region (anterior mandible)	Could not fit logistic regression			
Experience (junior)	1.38	“0.152 to 12.5”	0.762	ns
Side (bilateral)	Could not fit logistic regression			
Incision (sulcular flap)	1.14	“0.142 to 7.34”	0.891	ns
Osteotomy (bur)	Could not fit logistic regression			
Number of screws	Could not fit logistic regression			
Ian visibility (present)	9.33	“1.37 to 78.9”	0.023	*
Bad fracture	Could not fit logistic regression			
Bleeding	3	“0.139 to 26.0”	0.364	ns
**Early neurosensory disturbance**				
Age	0.991	“0.939 to 1.05”	0.745	ns
Gender (female)	3.2	“0.677 to 23.0”	0.174	ns
Medication (polymedication)	2	“0.0777 to 29.5”	0.617	ns
Systemic disease (ASA II)	1.47	“0.278 to 6.64”	0.624	ns
Smoking (present)	1.82	“0.395 to 8.38”	0.429	ns
Alcohol (present)	Could not fit logistic regression			
Tooth gap (three)	2.25	“0.0977 to 26.5”	0.528	ns
Type of anesthesia (sedation)	Could not fit logistic regression			
Region (anterior mandible)	Could not fit logistic regression			
Experience (junior)	1.38	“0.152 to 12.5”	0.762	ns
Side (bilateral)	0.49	“0.0251 to 3.10”	0.521	ns
Incision (sulcular flap)	1.82	“0.395 to 8.38”	0.429	ns
Osteotomy (bur)	2.07	“0.0986 to 16.7”	0.540	ns
Number of screws (one)	0.595	“0.0304 to 3.82”	0.642	ns
Ian visibility (present)	28	“5.22 to 224”	< 0.001	*
Bad fracture	Could not fit logistic regression			
Bleeding	1.63	“0.0791 to 12.2”	0.676	ns
Hematoma (present)	6.56	“0.755 to 48.1”	0.062	ns
**Infection**				
Age	1.05	“1.00 to 1.12”	0.054	ns
Gender (female)	0.524	“0.143 to 1.77”	0.305	ns
Medication (polymedication)	2	“0.0777 to 29.5”	0.617	ns
Systemic disease (ASA II)	1.6	“0.430 to 5.57”	0.463	ns
Smoking (present)	1.07	“0.291 to 3.65”	0.913	ns
Alcohol (present)	0.97	“0.135 to 4.47”	0.971	ns
Tooth gap (three)	1.07	“0.141 to 5.57”	0.939	ns
Type of anesthesia (sedation)	Could not fit logistic regression			
Region (anterior mandible)	1.97	“0.328 to 11.0”	0.436	ns
Experience (junior)	2	“0.385 to 11.5”	0.408	ns
Side (bilateral)	Could not fit logistic regression			
Incision (sulcular flap)	1.59	“0.455 to 5.42”	0.458	ns
Osteotomy (bur)	3.27	“0.397 to 22.1”	0.222	ns
Number of screws (one)	6.12	“1.58 to 24.4”	0.008	*
Ian visibility (present)	0.348	“0.0182 to 2.08”	0.335	ns
Bad fracture (present)	0.867	“0.0432 to 6.06”	0.9	ns
Bleeding	2.41	“0.307 to 14.1”	0.343	ns
Hematoma (present)	Could not fit logistic regression			
Early neurosensory disturbance (present)	Could not fit logistic regression			
**Dehiscence**				
Age	1.01	“0.968 to 1.06”	0.662	ns
Gender (female)	0.337	“0.0951 to 1.06”	0.073	ns
Medication (polymedication)	1.22	“0.0503 to 14.7”	0.88	ns
Systemic disease (ASA II)	0.725	“0.182 to 2.44”	0.62	ns
Smoking (present)	1.02	“0.307 to 3.18”	0.973	ns
Alcohol (present)	0.714	“0.101 to 3.20”	0.689	ns
Tooth gap (three)	0.701	“0.0945 to 3.48”	0.686	ns
Type of anesthesia (sedation)				
Region (anterior mandible)				
Experience (junior)	1.92	“0.432 to 9.09”	0.389	ns
Side (bilateral)	1.3	“0.317 to 4.63”	0.692	ns
Incision (sulcular flap)	1.43	“0.448 to 4.46”	0.535	ns
Osteotomy (bur)	6	“0.908 to 49.3”	0.063	ns
Number of screws (one)	2.61	“0.680 to 9.52”	0.146	ns
Primary stabilization (fair)	2.3	“0.470 to 11.0”	0.29	ns
Ian visibility (present)	0.629	“0.0897 to 2.76”	0.577	ns
Bad fracture (present)	0.653	“0.0328 to 4.48”	0.708	ns
Bleeding	3.92	“0.661 to 23.5”	0.118	ns
Hematoma (present)	Could not fit logistic regression			
Early neurosensory disturbance (present)	Could not fit logistic regression			
Infection (present)	6.22	“1.71 to 24.0”	0.006	*

Table 6, continuing the univariate logistic regression analysis from Table 5, evaluates factors affecting late postoperative complications, including age, sex, medication, ASA II systemic disease, smoking, alcohol use, tooth gap, anesthesia type, surgical region, surgeon experience, and more. Like in the analysis of early complications, some variables could not be assessed due to possible perfect separation or linear dependence. The analysis highlighted that the presence of infection significantly increased the risk of resorption (OR 7.15, *p* = 0.004), graft separation (OR 5.31, *p* = 0.020), and graft loss (OR 6.50, *p* = 0.011), although many factors did not significantly impact late postoperative complications.

**Table 6:** Logistic regression analysis for late postoperative complications

	**OR**	**95% CI**	**P**	**sig.**
Resorption				
Age	1.02	“0.978 to 1.07”	0.359	ns
Gender (female)	0.556	“0.166 to 1.75”	0.321	ns
Medication (polymedication)	1.66	“0.510 to 5.32”	0.392	ns
Systemic disease (ASA II)	Could not fit logistic regression			
Smoking (present)	1.22	“0.336 to 4.03”	0.751	ns
Alcohol (present)	0.786	“0.111 to 3.55”	0.775	ns
Tooth gap (three)	3.54	“0.722 to 17.2”	0.11	ns
Type of anesthesia (sedation)	Could not fit logistic regression			
Region (anterior mandible)	Could not fit logistic regression			
Experience (junior)	0.76	“0.138 to 3.61”	0.734	ns
Side (bilateral)	3.41	“0.947 to 12.1”	0.056	ns
Incision (sulcular flap)	0.81	“0.226 to 2.62”	0.731	ns
Osteotomy (bur)	6.63	“0.997 to 54.7”	0.051	ns
Number of screws (one)	2.94	“0.757 to 10.9”	0.107	ns
Primary stabilization (fair)	0.821	“0.107 to 4.32”	0.827	ns
Ian visibility (present)	Could not fit logistic regression			
Bad fracture (present)	1.96	“0.252 to 11.3”	0.464	ns
Bleeding	Could not fit logistic regression			
Hematoma (present)	Could not fit logistic regression			
Early neurosensory disturbance (present)	0.49	“0.0251 to 3.10”	0.521	ns
Infection (present)	7.15	“1.93 to 28.2”	0.004	*
**Graft separation**				
Age	0.982	“0.936 to 1.03”	0.444	ns
Gender (female)	1.16	“0.316 to 4.42”	0.822	ns
Medication (polymedication)	Could not fit logistic regression			
Systemic disease (ASA II)	0.854	“0.172 to 3.35”	0.83	ns
Smoking (present)	0.136	“0.00718 to 0.780”	0.065	ns
Alcohol (present)	Could not fit logistic regression			
Tooth gap (three)	3.33	“0.386 to 24.1”	0.23	ns
Type of anesthesia (sedation)	1.36	“0.186 to 6.62”	0.216	ns
Region (anterior mandible)	0.889	“0.112 to 5.05”	0.899	ns
Experience (junior)	1.43	“0.293 to 7.00”	0.651	ns
Side (bilateral)	2.49	“0.573 to 9.88”	0.198	ns
Incision (sulcular flap)	3.68	“0.991 to 15.5”	0.057	ns
Osteotomy (bur)	1.38	“0.0666 to 10.6”	0.785	ns
Number of screws (one)	1.84	“0.357 to 7.71”	0.424	ns
Primary stabilization (excellent)	0.322	“0.0154 to 2.68”	0.339	ns
Ian visibility (present)	2.08	“0.400 to 8.90”	0.339	ns
Bad fracture (present)	3.06	“0.384 to 18.3”	0.234	ns
Bleeding (present)	3.06	“0.384 to 18.3”	0.234	ns
Hematoma (present)	Could not fit logistic regression			
Early neurosensory disturbance (present)	Could not fit logistic regression			
Infection (present)	5.31	“1.28 to 22.2”	0.02	*
Dehiscence (present)	3.64	“0.906 to 14.4”	0.062	ns
Resorption (major)	1.47	“0.289 to 6.00”	0.608	ns
**Graft loss**				
Age	1.02	“0.973 to 1.08”	0.4	ns
Gender (female)	0.351	“0.0703 to 1.39”	0.155	ns
Medication (polymedication)	1.22	“0.0503 to 14.7”	0.88	ns
Systemic disease (ASA II)	1	“0.198 to 4.06”	> 0.999	ns
Smoking (present)	0.689	“0.138 to 2.76”	0.615	ns
Alcohol (present)	1.42	“0.193 to 6.89”	0.689	ns
Tooth gap (three)	1.89	“0.237 to 11.0”	0.497	ns
Type of anesthesia (sedation)	0.613	“0.0313 to 3.92”	0.66	ns
Region (anterior mandible)	5.33	“0.458 to 123”	0.193	ns
Experience (junior)	0.412	“0.0194 to 3.55”	0.46	ns
Side (bilateral)	1.5	“0.667 to 12.3”	0.134	ns
Incision (sulcular flap)	3	“0.771 to 12.9”	0.117	ns
Osteotomy (bur)	4.75	“0.562 to 33.3”	0.115	ns
Number of screws (one)	2.14	“0.410 to 9.28”	0.324	ns
Primary stabilization (excellent)	1.44	“0.290 to 7.87”	0.655	ns
Ian visibility (present)	Could not fit logistic regression			
Bad fracture (present)	Could not fit logistic regression			
Bleeding (present)	1.22	“0.0602 to 8.84”	0.862	ns
Hematoma (present)	Could not fit logistic regression			
Early neurosensory disturbance (present)	Could not fit logistic regression			
Infection (present)	6.5	“1.51 to 28.9”	0.011	*
Dehiscence (present)	2.67	“0.603 to 10.9”	0.174	ns
Resorption (major)	1.71	“0.332 to 7.24”	0.479	ns

### Adverse events and their management

Patients were followed up with extended antibiotic treatment for an additional 5 days in cases of hematoma, and dehiscence developed in 2 patients. In 13 patients 2–4 weeks postsurgery, infections characterized by facial swelling and restricted mouth opening were observed. As treatment, a 7-day course of systemic antibiotics (amoxicillin + clavulanic acid, 2 × 1 daily) was prescribed. Additionally, chlorhexidine mouthwash was recommended for oral care. In 16 patients, between 2 and 6 weeks after suture removal, incidents of flap opening and exposure of the graft were observed. The treatment involved curettage with curettes and smoothing of sharp bone edges using a low-speed piezosurgery device and a mounted diamond bur, all under continuous irrigation with sterile saline. The aim of this procedure was to promote secondary healing through reepithelization. In 13 of these patients, the dehiscence completely closed within 4 weeks following the intervention. However, in 3 patients, the opening persisted until the time of the implant operation.

Eight patients reported experiencing temporary paresthesia in the inferior alveolar nerve (IAN) on the side where the harvest procedure was performed. The symptoms and signs of nerve dysfunction spontaneously subsided within 2 to 8 weeks, and no cases of permanent nerve damage were observed. Exposure of the IAN during surgery significantly increased the risk of postoperative temporary paresthesia (OR = 28.00, 95% CI = 5.22 to 224; p = < 0.001). At clinical follow-up appointments, patients who reported no issues were considered neurosensorily stable, and their follow-up for this specific issue was concluded. For those who reported symptoms, a course of vitamin B complex (2 × 1 for 4 weeks) was initiated, and they were scheduled for follow-up visits at 1 month, 3 months, and 6 months to monitor their progress and response to treatment. In the long term, none of the patients reported any instances of permanent neurosensory disturbance. In ten patients, the graft was completely removed (Table 3). Of these, 8 underwent a second grafting procedure. The remaining 2 patients declined further surgical intervention and were managed with prosthetic treatments without dental implants.

## Discussion

Postoperative complications are unexpected or adverse outcomes that follow surgical intervention, and the severity of a complication is often related to the type of surgery performed. The development of strategies to prevent medical complications and the analysis of risk factors are fundamental for maintaining high-quality medical care [17]. This study specifically aimed to evaluate the risk factors and complication rates associated with mandibular ramus block grafting. Our findings indicate that the majority of patients who underwent alveolar crest augmentation experienced successful outcomes with minimal complications. However, it is crucial to recognize that complications may arise at various stages of the surgical process, including the intraoperative, early postoperative, and late postoperative phases. In autogenous grafting, complications can manifest during any of these periods. Identification and understanding of these potential complications are vital for improving surgical outcomes and patient care in oral and maxillofacial surgery.

While there are reports suggesting that patient age and sex do not significantly affect complication rates in patients after bone grafting [18], other publications have reported increased resorption in female patients [19, 20]. In our study, age did not significantly affect complication rates during any of the evaluated periods. Sex reached statistical significance only for intraoperative complications, specifically for IAN visibility, but not for early postoperative neurosensory disturbances, as confirmed by Fisher’s exact test. We hypothesized that anatomical differences between the sexes may contribute more significantly to IAN visibility. However, it’s important to acknowledge the limitations in our analysis, particularly in the context of univariate logistic regression, which may not fully capture the complex interplay of variables. Our results also indicated that commonly accepted risk factors such as smoking, which is widely regarded to negatively impact surgical success and wound healing [7, 21], were not statistically significant risk factors for complications. Similarly, variables such as alcohol consumption, medication use, or systemic illness did not appear to increase the risk of complications. These results may be relevant to the specific participant population and should be treated with caution. However, our findings are in line with those of a systematic review by Starch-Jensen T et al. [22], which also did not establish a relationship between postoperative complications and factors such as smoking habits, age, oral hygiene, or reason for tooth loss.

One of the most common morbidities associated with grafts harvested from the mandible is nerve damage, which has been reported at varying rates in different studies [23–25]. In a study by Khoury involving 3,328 patients, 7.35% of patients experienced temporary neurosensory disturbances due to inferior alveolar nerve (IAN) exposure, and persistent paresthesia lasting longer than a year was reported in 0.1% of patients (*n* = 4) [21]. The causes of nerve exposure include a weak external oblique line, osteotomy performed below the path followed by the nerve, and distal vertical osteotomy in the ascending ramus area, where the nerve runs close to the buccal cortex [21, 26]. In our study, we observed a significant increase in the risk of early neurosensory disturbance (OR = 28.00) when IAN exposure occurred. Although no long-term neurosensory disturbances were encountered in any of the patients included in our cohort, we recommend initiating pharmacological treatment earlier and informing the patient when IAN visibility is encountered.

Dehiscence is reported as one of the most common complications of surgery for the reconstruction of atrophic crests in some studies [27]. While the presence of dehiscence does not necessarily indicate definitive failure [28, 29], it is known to increase graft resorption [30]. It is recognized that dehiscence encountered in guided tissue regeneration procedures can reduce bone formation by up to sixfold [31]. The causes of dehiscence may include the width of the keratinized mucosa, flap thickness, flap tension, vestibular depth, and the size of the defect in the alveolus [32]. Parallel to the literature [33], our experience also indicates that the most significant cause is the inability to close the flap without tension. The use of matrix sutures and extraoral pressure dressings are also recommended methods for preventing the separation of wound edges [10]. Dehiscence and infection are distinct concepts associated with different clinical outcomes, and they should be reported separately in studies [21, 28]. For the treatment of this complication, vigorous gargling with antibiotics and antiseptics for up to 3 weeks is recommended. However, if dehiscence and the consequent exposure of the graft worsen, removal of the bone may also be necessary [30]. In our study, infection was the most critical factor leading to graft loss.

Block grafts, known to better maintain their volume than particle grafts [34], often experience graft resorption, which varies among patients [35, 36]. Graft resorption rates in the literature vary between 25% and 60% [37, 38]. Although resorption in blocks harvested from the ramus is known to occur less often due to reasons such as the intramembranous nature of the bone, the corticocancellous ratio [39], and better adaptation to the recipient site [40], in some cases, excessive resorption necessitates secondary bone grafting [41]. According to a study by Nyström et al., the majority of resorptions occur in the first 6 months, with no evidence of continuation after 12 months [20]. In our study, the average resorption rate measured during implant placement between 4 and 8 months was 1.09 mm (SD = 0.89). One of the suggested methods to address resorption is overcorrection using larger grafts [42], but this can increase morbidity at the donor site and the likelihood of dehiscence at the recipient site. While there are publications reporting positive effects of contour augmentation using membrane and/or particle grafts in reducing resorption [43, 44], prospective randomized controlled trials in the literature also indicate that these applications may increase the likelihood of complications [45]. In cases where graft-membrane application is used, dehiscence development may require curettage of the exposed membrane and graft. According to our study’s results and clinical experience, the most significant factor in resorption was infection at the recipient site (OR = 7.15).

Graft failure and subsequent removal are complications that essentially resume the entire process, leading to unfavorable circumstances for both the patient and the surgeon. In a systematic review evaluating 23 studies between 2015 and 2020, the major infection rate was found to be 1.6% [28]. In our study, 14.29% of the patients experienced graft loss (*n* = 10). According to Buser, a stepwise approach reduces the likelihood of complications such as dehiscence, membrane exposure, and graft loss. Based on our observations, the success of the autogenous grafting procedure depends on the equal importance of each stage, from the initial incision to flap closure.

The stability of the bone graft is a precondition for the success of crest augmentation, as outlined in the PASS principles by Wang et al. [46]. The use of titanium mini screws for rigid fixation of the block graft to the recipient area has been found to be essential for successful graft success and for preventing fibrous growth between the autogenous block graft and the host. In this study, block grafts were fixed with two titanium mini screws and shaped to enhance graft adaptation [47]. One of the primary reasons for the separation of an uninfected graft during implant surgery could be inadequate adaptation of the graft or insufficient bone-graft contact during the initial operation [4]. The preparation of the recipient site [48] and the precise adaptation of the bone block [4] are critical for graft revascularization and success. Close contact between the graft and the bone prevents micromovement of the graft and associated vascular damage [49]. While decortication at the recipient site is suggested to enhance revascularization [50, 51], the scientific basis for this recommendation is weak. To prevent this separation, one method recommended in the literature is not removing osteosynthesis screws that do not interfere with implant placement but rather leaving them in place [52, 53]. When planning the placement of screws, if the graft is allowed, planning them in areas that will not be drilled during implant surgery could yield better clinical outcomes. Another suggestion is to remove osteosynthesis screws as late as possible during the preparation of the implant site with a drill. Additionally, based on the results of this study, we believe that using two screws instead of one may have a positive impact on healing, despite the increased risk of infection (OR = 6.12).

Our study has many strengths, such as the detailed classification and analysis of complications in alveolar grafting, practical solutions drawn from the literature and experience. These findings are crucial for improving clinical practices and patient care, emphasizing the importance of thorough patient selection, preoperative assessment, and informed consent to mitigate risks and manage potential complications effectively. This study has several limitations due to its retrospective nature, potentially leading to incomplete data and unaccounted variations in complication rates. The absence of standardized complication records in surgical notes is a recognized shortcoming. The one-year follow-up period, while providing valuable initial information, is relatively short and may not adequately capture long-term outcomes and complications, a limitation that may affect the longevity and stability of grafting results and dental implant success. Furthermore, while the sample size was sufficient for initial analysis, it may be too small for the more complex multivariate analysis required to thoroughly explore the interactions among variables like implant types, surgical techniques, and surgeon experience levels. Although the low frequency of severe complications could be considered a limitation, prospective studies with larger patient cohorts are needed to ascertain the true incidence of complications and minimize biases.

## Conclusion

This study was a comprehensive analysis evaluating donor site complications after autogenous bone grafting and identifying risk factors. Analysis of a consecutive cohort of 70 patients who underwent autogenous ramus grafting at a university teaching hospital in Turkey showed optimal outcomes and a low rate of major complications. This information should help clinicians advise patients requiring autogenous grafting and enable them to make informed decisions about their management.

### Declarations section

## Data Availability

No datasets were generated or analysed during the current study.
